# Patient Utilization of Online Information and its Influence on Orthopedic Surgeon Selection: Cross-sectional Survey of Patient Beliefs and Behaviors

**DOI:** 10.2196/22586

**Published:** 2022-01-19

**Authors:** Victor Hoang, Amit Parekh, Kevin Sagers, Trevor Call, Shain Howard, Jason Hoffman, Daniel Lee

**Affiliations:** 1 Valley Hospital Medical Center Las Vegas, NV United States

**Keywords:** orthopedics, practice management, physician selection, internet reviews, patient decision, practice, patient online review, social media, physician perception, patient choice, health literacy

## Abstract

**Background:**

Patient attitudes and behavior are critical to understand owing to the increasing role of patient choice. There is a paucity of investigation into the perceived credibility of online information and whether such information impacts how patients choose their surgeons.

**Objective:**

The purpose of this study was to explore the attitudes and behavior of patients regarding online information and orthopedic surgeon selection. Secondary purposes included gaining insight into the relative importance of provider selection factors, and their association with patient age and education level.

**Methods:**

This was a cross-sectional study involving five multispecialty orthopedic surgery groups. A total of 329 patients who sought treatment by six different orthopedic surgeons were asked to anonymously answer a questionnaire consisting of 25 questions. Four questions regarded demographic information, 10 questions asked patients to rate the importance of specific criteria regarding the selection of their orthopedic surgeon (on a 4-point Likert scale), and 6 questions were designed to determine patient attitude and behaviors related to online information.

**Results:**

Patient-reported referral sources included the emergency room (29/329, 8.8%), friend (42/329, 12.8%), insurance company (47/329, 14.3%), internet search/website (28/329, 8.5%), primary care physician (148/329, 45.0%), and other (34/329, 10.3%). Among the 329 patients, 130 (39.5%) reported that they searched the internet for information before their first visit. There was a trend of increased belief in online information to be accurate and complete in younger age groups (*P*=.02). There was an increased relative frequency in younger groups to perceive physician rating websites to be unbiased (*P*=.003), provide sufficient patient satisfaction information (*P*=.01), and information about physician education and training (*P*=.03). There was a significant trend for patients that found a surgeon’s website to be useful (*P*<.001), with the relative frequency increased in younger age groups.

**Conclusions:**

This study shows that insurance network, physician referrals, appointment availability, and office location are important to patients, whereas advertising and internet reviews by other patients were considered to be not as helpful in choosing an orthopedic surgeon. Future studies may seek to identify obstacles to patients in integrating online resources for decision-making and strategies to improve health-seeking behaviors.

## Introduction

Health literacy is a complex concept, defined by both the Institute of Medicine and the World Health Organization as incorporating cognitive and social skill sets that are distilled through patient experiences and are necessary to obtain, understand, and apply information to make appropriate health decisions [1,2]. Health organizations have underscored the importance of health literacy as an essential component of patient-centered care [3,4]. Consequently, a rich body of literature established factors that influence health care choices [4-8] and investigated trends in health care consumerism [9-11].

Online tools and information are postulated to disrupt the traditional patient-physician relationship and traditional metrics of health care assessment with the expanded use of social media and physician rating websites (PRWs) [9,12-15]. Physician websites, social media venues, and online review sites are the most common spaces in which patients can discover information about physicians and their practices [16]. Previous studies sought to investigate online patient behavior and classify the information posted online by patients [11,13,17-19]. The rating scales on PRWs were found to be inaccurate and with significant limitations; as such, concerns regarding malignment of consumer satisfaction and quality were raised because health care incentives are not aligned as in other consumer industries [17,20,21]. Roughly 59% of US respondents indicated that they believed that the information on PRWs is either somewhat or very important [22], despite the documented disparity between conventional quality metrics and crowd-sourced online reviews [20,21,23,24]. Thus, it remains unclear why patients use these platforms and if this information influences their behavior [4].

The impact of misinformed or uniformed patients is consequential [25-30]. Limited health literacy has been associated with low patient satisfaction, worse patient outcomes, and higher costs [31,32]. In orthopedic surgery, there is a unique form of health literacy and a more sophisticated skill set required for making informed decisions [33-35]. Decision-making has been found to not be strictly rational but is rather a complex and heterogenous process that is distilled through patient preferences, values, and social influences [5,36]. Improved understanding of these influences on patient decision-making may identify actionable opportunities to practice patient-centered care. To our knowledge, there is a paucity of investigations eliciting how patient attitudes and behaviors related to information online influence provider selection factors. Considering that such information may be of low quality and inaccurate [23,37], it is important to explore if online research alters a patient’s decision-making for provider selection. 

Accordingly, the aim of this study was to define the internet sources that patients are using to research their orthopedic surgeons and to quantify the importance placed on those findings. In addition, we investigated the demographic variables that may influence the reliance on internet websites, and further aimed to define the importance of other variables involved in choosing an orthopedic surgeon. The purpose of this study was to explore the attitudes and behavior of patients regarding online information and its influence on establishing care with an orthopedic surgeon. Secondary purposes included a description of the relative importance of provider selection factors, and their association with patient age and education level.

## Methods

We performed a cross-sectional survey of patients at orthopedic offices in Las Vegas, Nevada. The study group included six orthopedic surgeon practices screening patients in their clinics. Subspecialties included were foot and ankle, hand, spine, and sports medicine. The surveys were completed by patients that were seen at the clinics over the course of 3 months. This study was approved by OptiWest institutional review board. Strengthening the Reporting of Observation studies in Epidemiology (STROBE) and Statistical Analyses and Methods in the Published Literature (SAMPL) reporting guidelines were followed during the study design and manuscript preparation to ensure methodologic quality and transparent reporting [38,39].

Consent from each patient was obtained before participation. The survey was confidential and anonymous, with no identifiers linked to individual responses. All participants completed the survey.

The survey consisted of seven questions, which aimed to gauge patient opinion and define patient behavior (see Multimedia Appendix 1). The survey asked patients to report their demographics, attitudes, and behaviors. Three questions documented patient demographics: patient age, education level, and frequency of internet use. One question prompted patients to rate specific orthopedic surgeon selection criteria [6-8,36] on a 4-point Likert scale ranging from 1 defined as “not important” to 4 defined as “very important.” Two questions polled patient opinion regarding internet patient reviews and if patient satisfaction equates to a successful treatment outcome. One question assessed the patient’s use of websites prior to their clinic visit. The participants completed their surveys in person and responses were kept anonymous. Notably, the survey is not a validated questionnaire of a measure of a specific outcome but rather represents a survey of questions. This article reports the results of the descriptive analysis of the responses for an exploratory investigation into patient beliefs, behaviors, and trends.

Respondents were grouped into the following age ranges: 18-25, 26-35, 36-45, 46-55, 56-65, and 76-85 years. Respondents were stratified based on their highest level of education: elementary/middle school, high school, some college, bachelor’s degree, master’s degree, and doctoral degree. A trained medical assistant or research assistant explained each question to the participants while administering the survey.

Basic descriptive statistics were analyzed using MedCalc Software. Ordinal Likert-scale data are reported using median for central tendency and frequencies, and Kendall τ was used to analyze associations. Associations are reported as the correlation coefficient with a precision estimate (95% CI) [40]. The Cochran-Armitage test was used for analysis of categorical variables [41,42], which is considered to be more powerful than the *χ^2^* test to assess trends in proportions and frequencies. The statistical significance level was set at *P*<.05.

## Results

**Figure 1:**
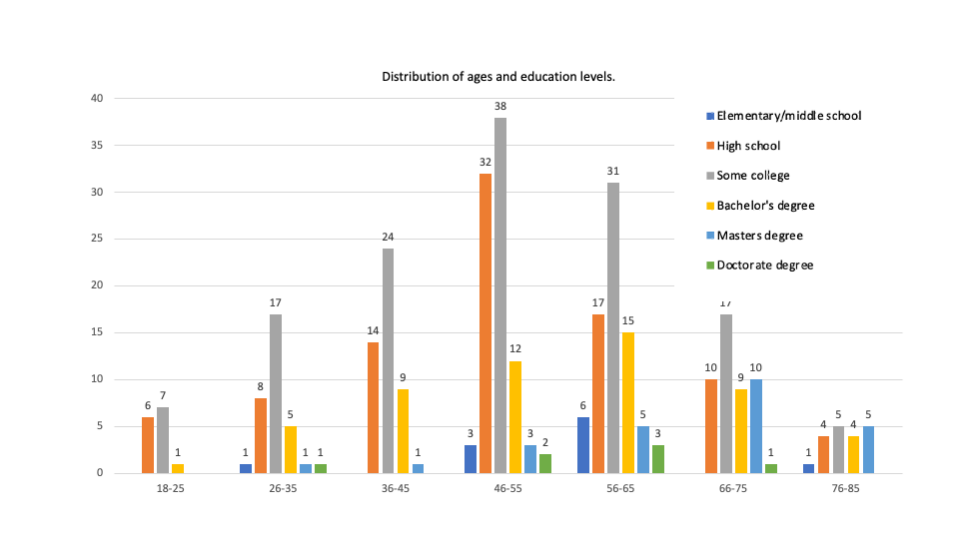
Respondent age and highest education stratification of all participants (N=329).

Between July 2017 and August 2017, all 329 patients that were administered the survey completed the survey. Table 1 delineates the distribution of patients that completed the survey according to the subspecialty of the orthopedic surgeon they were consulting. The majority of patients reported daily baseline internet use (227/329, 69.0%), followed by 2-3 times per week (23/329, 7.0%) and 4-5 times per week (20/329, 6.1%). The histogram of the number of patients that responded according to age group and stratified by the highest education level is shown in Figure 1.

**Table 1:** Survey participants stratified by orthopedic subspecialty (N=324; subspecialties were not documented by 5 patients).

Subspecialty	Patients, n (%)
Spine	150 (46.3)
Sport	119 (36.7)
Hand	33 (10.2)
Foot and ankle	22 (6.8)

The patient-reported referral source was the emergency room (29/329, 8.8%), friend (42/329, 12.8%), insurance company (47/329, 14.3%), internet search/website (28/329, 8.5%), primary care physician (PCP; 148/329, 45.0%), and other (34/329, 10.3%). Among the 329 patients, 130 (39.5%) reported that they had searched the internet for information about the surgeon before their first visit. The majority of these patients had visited the surgeon’s website (63/130, 48.5%), followed by the website of the office or surgical group (35/130, 26.9%). Other websites visited included webmd.com (34/130, 26.2%), yelp.com (26/130, 20.0%), healthgrades (21/130, 16.2%), ratemd.com (20/130, 15.4%), and the Nevada medical board website (7/130, 5.4%).

**Figure 2:**
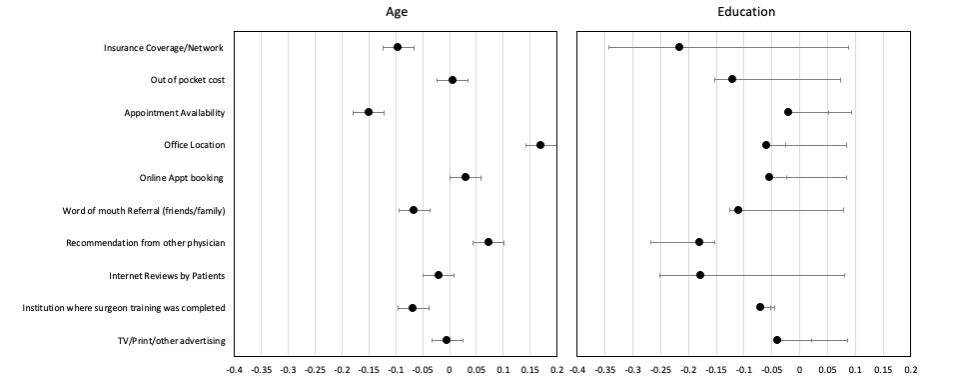
Relative importance of orthopedic surgeon selection factors, and their associations with patient age and education level.

The ranking of important factors in selecting the orthopedic surgeon is displayed in Figure 2 as well as the association of these factors with age and level of education. Patient age was significantly associated with office location (*P*=.05), physician recommendation (*P*<.001), internet reviews (*P*<.001), and advertising sources (*P*=.01). Patient education level was significantly associated with out-of-pocket costs (*P*=.05), availability (*P*<.001), office location (*P*<.001), online appointment booking (*P*=.004), surgeon training (*P*=.002), and advertisement sources (*P*<.001). Patients reported insurance coverage (260/329, 79.0%), out-of-pocket costs (217/329, 66.0%), availability (184/329, 55.9%), and recommendation by another physician as “very important” (score of 4). Surgeon advertising was rated 1 (not important) by 204 (62.0%) of the 329 patients. The frequency at which internet reviews were deemed to be important ranged between 21% and 29% in each category. The institution where the surgeon trained was only deemed to be very important for 82 (24.9%) and as moderately important for 99 (30.1%) of the 329 respondents.

The attitudes of patients toward online information are summarized in Table 2. The highest frequency of patients indicated that they found the surgeon’s website to be useful. Among the factors included in the questionnaire, the lowest number of patients indicated that online information is accurate and complete. There were no significant associations found between patient education groups in regard to their online information or PRW beliefs.

**Table 2:** Attitude toward online information (“is it important?”) (N=329).

Question	Yes, n (%)	Age *P* value	Education *P* value
Online information is accurate and complete	40 (12.2)	.02	.99
PRW^a^ is unbiased	131 (39.8)	.003	.32
PRW has complicated rate information	95 (28.9)	.09	.21
Ongoing or previous litigation claims	80 (24.3)	.24	.52
PRW shows patient satisfaction	138 (41.9)	.01	.95
PRW indicates education and training	147 (44.7)	.03	.58
Surgeon website useful	189 (57.4)	<.001	.18

^a^PRW: physician rating website.

**Figure 3:**
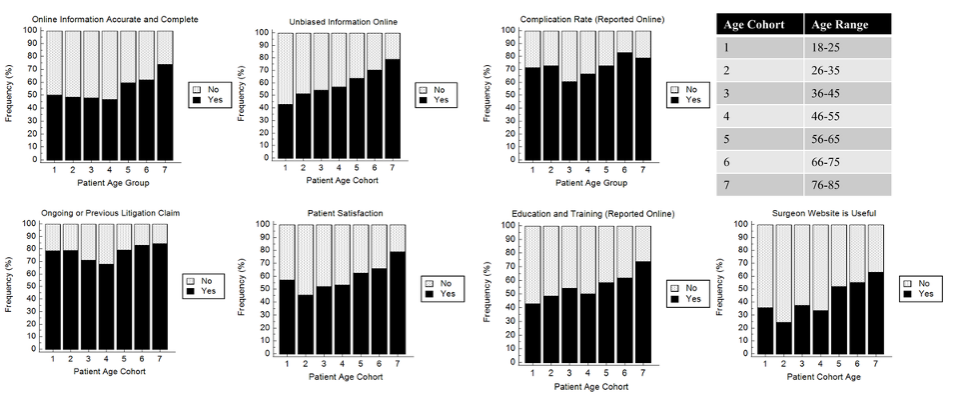
Trends in relative frequency of patient perceptions. Each subgraph, further categorized by age, shows if a specific factor influences the patient’s selection of orthopedic surgeons.

Significant trends were found in beliefs regarding online information and PRWs between age groups (Figure 3). There was a trend of increased belief in online information to be accurate and complete in the younger age groups (*P*=.02). There was an increased relative frequency in younger groups to perceive PRWs to be unbiased (*P*=.003), provide sufficient patient satisfaction information (*P*=.01), and information about physician education and training (*P*=.03). There was also a significant trend for patients that found the surgeon’s website to be useful (*P*<.001), with the relative frequency increased in younger age groups.

## Discussion

### Principal Findings

Only 28 of the 329 patients (8.5%) that completed the survey selected their orthopedic surgeon using internet search/websites. Notably, 205 (62.3%) patients were referred to their orthopedic surgeon from health care–related sources (emergency room, insurance company, and PCP), with the highest percentage of patients (148/329, 45.0%) referred by their PCP. Correspondingly, the data reflected the generally low importance of patient-oriented advertisements, with 204 patients (62.0%) giving this factor a rating of 1 (not important). Our data indicate that patients are value-oriented, and rated insurance coverage (260/329, 79.0%) and out-of-pocket costs (189/329, 57.4%) as very important factors. Only 130 of the 329 patients (39.5%) conducted an internet search prior to their first visit. Notably, there were significant trends observed for younger patient groups believing online information to be accurate and complete, as well as having more favorable attitudes toward PRWs in providing sufficient and unbiased information (Figure 3).

Despite the rapid expansion of online information available to patients, our data indicate that patients do not use this information to actively engage in their care. This conclusion is in support of previously published findings [43,44]. Patients also did not seek to learn about provider medical knowledge, litigation, or patient satisfaction. Patient satisfaction was purported to be a quality-of-care surrogate metric, considering the complex interplay of social, demographic, cultural, and cognitive factor interactions that influence satisfaction. The multidimensional assessment of quality was lost and deemed inappropriate [21]. Rothenfluh et al [45] suggested that one reason for this may be the perceived inability to assess physician quality even if informed by available information online, demonstrating that patients differ in decision-making between hotel selection and provider selection due to reduced trust in incorporating online information about physicians. Nevertheless, we found that 40% of patients utilized internet sources for information before their clinic visit compared to only 24% of patients reporting such use among those visiting an outpatient orthopedic clinic surveyed in 2002 [46]. Integration of online information is likely lagging in utilization, and future research should seek to delineate the causal factors or barriers.

Our data imply that surgeons should focus on their relationships with community physician referral sources. This was previously highlighted in a study on referrals to plastic surgeons [47] showing that 82% of patients felt that a recommendation from another physician was very important to moderately important, which was a statistically significant result across all age groups. Important factors influencing the choice of a foot and ankle surgeon were identified as insurance network and recommendations (family, friend, physician) [4]. Our data provide corroboratory support to these factors as important influences on patient decision-making. Further, the external validity of the findings can be compared among studies. In another study, important factors for patient selection of their surgeon and hospital for total joint arthroplasty were ranked on a 5-point Likert scale [6]. All three aforementioned reports [4,46,47] indicated that professional reputation is critical. Similarly, recommendations by other physicians and insurance companies had a significant impact on women selecting their obstetrician/gynecologist [44]. Future studies should evaluate whether there is a difference between how much patients weigh primary care versus urgent/emergent care referrals, other orthopedic surgeons’ opinions, and other medical providers in the community.

The correlation patterns found in this study were surprising and warrant attention. Age and education level have been proposed to influence health literacy, noting that patients with a graduate degree are 130 times more likely to have adequate health literacy (*P*=.01) [48]. Less than college-level education was previously identified as an independent predictor of limited musculoskeletal health literacy with a relative risk of 1.40 [49]. Our data demonstrated different statistically significant associations that had nonconsequential effect sizes. Importantly, this is not the first study to report younger age to be significantly associated with increased use and increased perceived usefulness of online information [50,51].

This survey was not without its limitations. The survey was administered to a convenience sample of limited size. Thus, the sample size of patients is underpowered, although the study was open to all patients at a large private practice setting in an anonymous fashion. Similarly, the selection bias within our sample cannot be ascertained. Another major flaw is the lack of a comparison group, which adds further sample bias. The surveys were also administered over time, with variability in practice settings, providers, and survey administrators, which could introduce recording and recall bias. Although we were unable to precisely determine the population percentage captured, the survey was administered in multiple locations and to multiple specialties of orthopedics to increase sample diversity. Our survey is not a standardized or validated questionnaire; thus, response bias may have been introduced. Nevertheless, our goal was to describe a macroscopic phenomenon rather than to deduce a causative process.

### Conclusions

This study shows that insurance network, physician referrals, appointment availability, and office location are important to patients, whereas advertising and internet reviews by other patients are not as helpful in choosing an orthopedic surgeon. Our data do not support consensus ideas regarding consumer autonomy and patient agency in health care. Future studies may seek to identify obstacles to patients in integrating online resources for decision-making and strategies to improve health-seeking behaviors.

## Appendix

Multimedia Appendix 1 Patient survey.

## Abbreviations

PCP: primary care physician
PRW: physician rating website
SAMPL: Statistical Analyses and Methods in the Published Literature
STROBE: Strengthening the Reporting of Observation studies in Epidemiology
