# Supplementary material for: Patient Utilization of Online Information and its Influence on Orthopedic Surgeon Selection: Cross-sectional Survey of Patient Beliefs and Behaviors
Source: JMIR Form Res. 2022 Jan 19;6(1):e22586. doi: 10.2196/22586 (PMC8811697; doi:10.2196/22586)
Supplement: Multimedia Appendix 1 [file formative_v6i1e22586_app1.docx]

Please answer the questions below and return this sheet to the medical assistant or resident physician. Thank you for your participation.

1. What is your age?

18-25  26-35  36-45  46-55  56-65  66-75  76-85  85+

1. Please indicate the level of education you have completed:

Elementary/Middle School  High School  Some College  Bachelor’s Degree  Masters Degree  Doctorate Degree

1. How often do you use the internet?

Daily  4-5 times/week  2-3 times/week  weekly

1. How important to you are the following when choosing an orthopedic specialist?

|  | Not Important 1 | Slightly Important 2 | Moderately Important 3 | Very Important 4 |
| --- | --- | --- | --- | --- |
| Medical Knowledge |  |  |  |  |
| Surgical Skill |  |  |  |  |
| Bedside Manner |  |  |  |  |
| Insurance Network Compatibility |  |  |  |  |
| Out of Pocket Cost |  |  |  |  |
| Availability |  |  |  |  |
| Word of Mouth Referrals from Friends/Family |  |  |  |  |
| Recommendations from other Physicians |  |  |  |  |
| Internet Reviews by Patients |  |  |  |  |
| Institution where Surgeon’s Training was Completed |  |  |  |  |
| TV/Print/Other Advertising |  |  |  |  |

1. Please indicate any/all websites you have used to obtain information on the orthopedic specialist you are seeing today:

| RateMD.com | Healthgrades.com | Yelp.com | Facebook.com |
| --- | --- | --- | --- |
| WebMD.com | Vitals.com | ZocDoc.com | ConsumerReports.com |
| Twitter.com | Website of office/  surgical group | | Surgeon's website |

1. Do you feel Patient Satisfaction is equivalent to a successful treatment outcome?

Yes  No

1. Do you feel that websites offering patient reviews regarding orthopedic surgeons provide information on the following:

| Accurate/complete information on the surgeon and practice | Yes  No |
| --- | --- |
| Unbiased Reviews | Yes  No |
| Treatment Complication Rates | Yes  No |
| Ongoing or Previous Litigation Claims | Yes  No |
| Data regarding Patient Satisfaction | Yes  No |
